# Diagnostic efficacy of long non-coding RNA *MALAT-1* in human cancers: a meta-analysis study

**DOI:** 10.18632/oncotarget.21013

**Published:** 2017-09-18

**Authors:** Yan Chen, Zhenzhou Xiao, Minhua Hu, Xiaoli Luo, Zhaolei Cui

**Affiliations:** ^1^ Laboratory of Biochemistry and Molecular Biology Research, Fujian Provincial Key Laboratory of Tumor Biotherapy, Department of Clinical Laboratory, Fujian Cancer Hospital and Fujian Medical University Cancer Hospital, Fuzhou, Fujian, China

**Keywords:** MALAT-1, cancer, diagnosis, meta-analysis

## Abstract

Metastasis-associated lung adenocarcinoma transcript 1 (*MALAT-1*) is one kind of long non-coding RNAs (lncRNAs) that has been recognized as a hallmark of the onset and development of several carcinomas. This study seek to meta-analyze the overall diagnostic efficacy of elevated *MALAT-1* expression profile for human cancers. Studies on the diagnostic performance of *MALAT-1* in cancers were retrieved by searching the online databases. The combined effect sizes were summarized using a bivariate meta-analysis model. Impacts of publication bias on the pooled effect sizes were assessed using “Duval and Tweedie nonparametric trim and fill method”. Sensitivity analysis and meta-regression test were applied to deeply trace the heterogeneity sources among eligible studies. A total of 14 studies with 1342 cancer cases were included. The combined effect sizes showed that *MALAT-1* expression profiling conferred an estimated sensitivity of 0.69 (95% CI: 0.62–0.75) (*I*^2^ = 84.01%, *P* < 0.001), specificity of 0.85 (95% CI: 0.79–0.90) (*I*^2^ = 87.95%, *P* < 0.001) and AUC (area under curve) of 0.83 in distinguishing cancer patients from noncancerous contrasts. Moreover, stratified analysis depending on cancer type manifested that elevated *MALAT-1* harbored a promising efficacy in the diagnosis of pulmonary tumors (AUC = 0.90), digestive system tumors (AUC = 0.84), gynecologic cancers (AUC = 0.84) and nasopharyngeal carcinoma (AUC = 0.84), particularly in confirming the subtype of squamous carcinoma (AUC = 0.91) and non-small cell lung carcinoma (AUC = 0.88) in lung cancer. Other analyses based on test matrix and ethnicity also presented robust results. Collectively, elevated *MALAT-1* could be developed as an auxiliary molecular marker to aid in cancer diagnosis.

## INTRODUCTION

Cancer is now becoming a global burden that has increased massive economic and social pressures around the world. According to the global cancer statistics data in 2012, nearly 14.1 million new cancer cases and 8.2 million cancer deaths were reported worldwide [1]. Early detection and treatment remains the major effective approach to help the cancer patients obtain favorable clinical outcomes. The current bloody tumor biomarkers are far from enough to satisfy the diagnosis of cancer in clinic owing to a relative low diagnostic efficacy.

In human beings, the majority of the genome is transcribed, yet only 2% accounts for protein-coding exons [2]. Long noncoding RNA (lncRNA) is an important population of the non-coding RNAs (ncRNAs) with a sequence longer than 200 nucleotides [3]. Evidence suggests that the lncRNAs can be classified as tumor suppressor genes or oncogenes according to their functions and expression pattern in tumoral tissues [4]. Metastasis-associated lung adenocarcinoma transcript 1 (*MALAT-1*) is one kind of nuclear lncRNAs that implicates in a spectrum of biological processes in vertebrate cells [5, 6]. *MALAT-1* is overexpressed in multiple types of human malignancies, such as the pulmonary cancers, digestive system tumors and genitourinary cancers, and so forth [7–20]. Notably, recent clinical studies have highlighted that *MALAT-1* could be rated as a promising biomarker to aid in cancer diagnosis and prognosis [7–22]. The clinical value of *MALAT-1* in predicting metastasis and/or survival in cancers has been documented by some meta-analysis studies [21, 22]. To our knowledge, no evidence-based meta-analyses have been reported on the overall diagnostic performance of *MALAT-1* for carcinomas. In order to elucidate the global diagnostic efficacy of *MALAT-1* in identification of cancers and provide valid evidences, we conducted this meta-analysis according to standard statistical methods. Our data may help to better understand the clinical value of elevated *MALAT-1* in the diagnosis of various cancers.

## RESULTS

### Study search and inclusion

**Figure 1:**
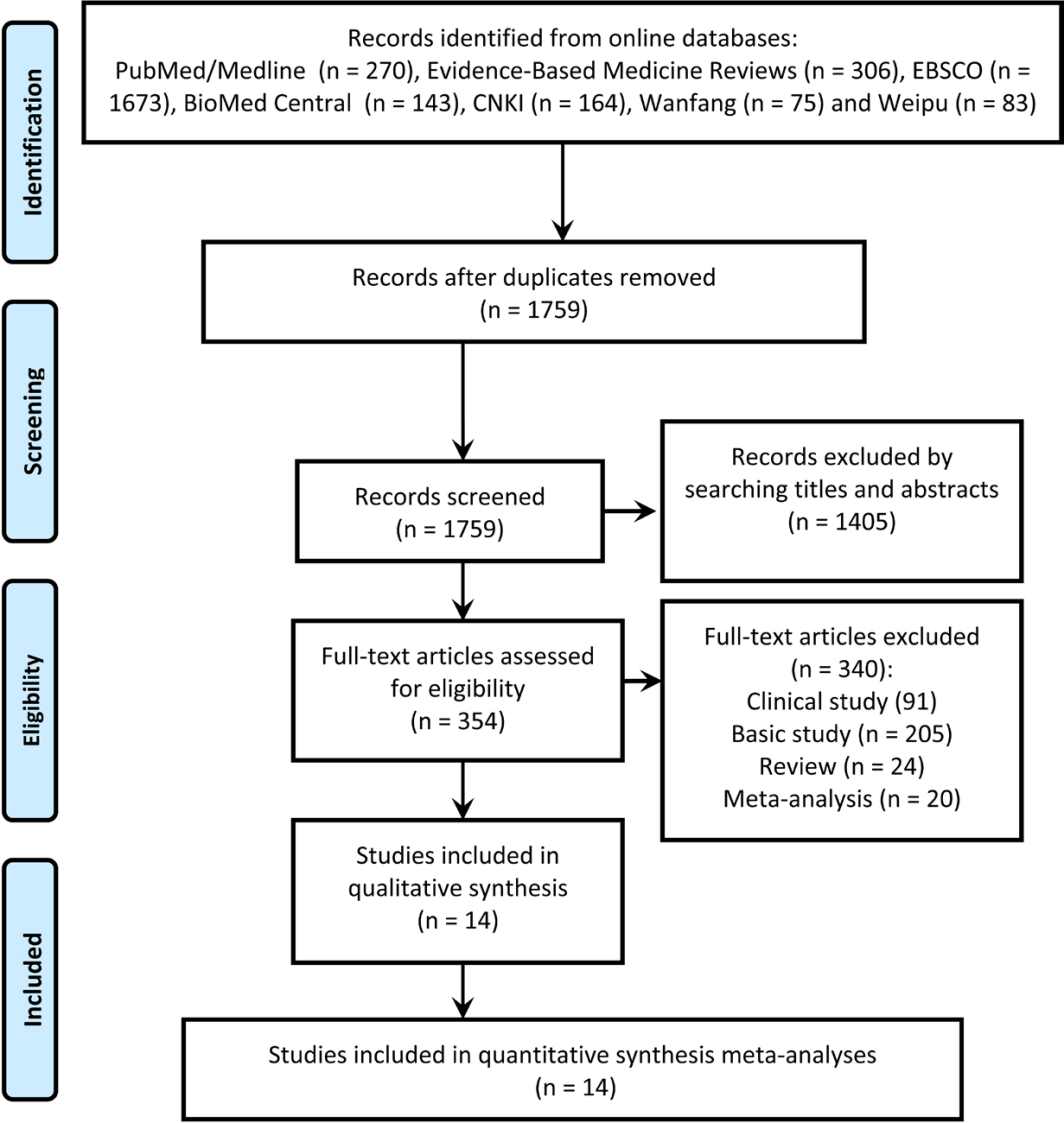
Study selection according to the procedures of the PRISMA diagram

The search of relevant articles was undertaken following the procedures of the PRISMA diagram (Figure 1). Briefly, a total of 1759 records in line with the search strategy were initially included from the electronic databases following an elimination of duplicates. Then, titles and abstracts of the records received detailed evaluation and 1405 of them were eliminated due to the status that not fitting the topic of our study. The following 354 records underwent full-text identification, and 24 of them were identified as reviews, 205 were basic studies, 91 were clinical studies, 20 were meta-analyses, and thus were all discarded. At last, 14 studies [7–20] assessed the diagnostic utility of up-regulated *MALAT-1* in cancer were included in the statistical analysis.

### Study characteristics and quality

All essential data were collected from 14 studies, comprising a combined population of 1342 cancer patients and 1189 noncancerous controls. All cancer patients had a definite diagnosis based on histopathological examination. All samples were obtained by surgical operation or biopsy prior to other therapies, and the specimen sources were from plasma [8, 12, 15, 18], serum [14, 16, 17, 19, 20], urine [10] and tissue [7, 9, 13]. The included neoplasm types involved lung cancer [8, 16–18], colorectal cancer [7], prostate cancer [10], breast cancer [19], ovarian cancer [12], pancreatic cancer [9], hepatocellular carcinoma [15], bladder cancer [14] and nasopharyngeal carcinoma [20]. Among the 14 publications, 13 studies directly reported the estimated sensitivity and specificity, 1 study showed the original data of TP (true positive), FP (false positive), FN (false negative) and TN (true negative) [8], and 1 study provided indirect data [19]. The levels of *MALAT-1* were determined based on the approach of quantitative real-time polymerase chain reaction (qRT-PCR), and that *GAPDH* [8, 9, 11, 13, 14, 16, 18–20], *β-actin* [7, 12, 15, 17], *HPRT190* [8], RPLP0 [8], or *PSAKIT* [10] were utilized as the reference genes. The essential data of all included studies are summarized in Table 1.

**Table 1:** Main features of the included studies

Study	Year	Area	Cancer type	Control type	Patient/Control size	Test matrix	Method	Reference gene	Cut-off value	AUC	Sensitivity/Specificity	QUADAS
Wen [18]	2016	China	Lung cancer	HC	84/60	Plasma	qRT-PCR	GAPDH	0.03	0.70	0.58/0.82	5
			AdCa		34/60					0.81	0.76/0.83	
			SqCC		26/60					0.93	0.92/0.82	
			SCLC		24/60					0.98	0.96/0.93	
Shi [17]	2016	China	Lung cancer	Non-cancer	60/92	Serum	qRT-PCR	β-actin	0.62	0.95	0.87/0.94	4
Chang [7]	2008	China	Colorectal cancer	HC	47/53	Tissue	qRT-PCR	β-actin	3.215	0.75	0.72/0.75	4
Wang [10]	2014	China	Prostate cancer	Biopsies negative	85/133	Urine	qRT-PCR	PSAKIT	MALAT-1 score: 95.0	0.688	0.82/0.63	5
				Biopsies negative	23/71					0.742	0.65/0.53	
				Biopsies negative	81/135					0.661	0.65/0.67	
				Biopsies negative	26/63					0.67	0.62/0.56	
Miao [19]	2016	China	Breast cancer	Non-cancer	78/40	Serum	qRT-PCR	GAPDH	Unclear	0.83	Extracted inderectively	4
Chen [12]	2016	China	Ovarian cancer	HC	94/47	Plasma	qRT-PCR	β-actin	0.617	0.88	0.72/0.89	5
Konishi [15]	2016	Japan	Hepatocellular carcinoma	Non-cancer	88/28	Plasma	qRT-PCR	β-actin	1.60	0.66	0.51/0.89	6
Han [13]	2016	China	Endometrial cancer	Adjacent cancer tissue	104/104	Tissue	qRT-PCR	GAPDH	6.445	0.73	0.45/0.82	4
Liu [9]	2014	China	Pancreatic cancer	Non-cancer	45/25	Tissue	qRT-PCR	GAPDH	0.1035	0.69	0.78/0.60	6
Weber [8]	2015	Germany	NSCLC	Non-cancer	45/25	Plasma	qRT-PCR	GAPDH HPRT1 RPLP0	0.41	0.79	Based on different cut-off settings	6
			Lung AdCa		21/25				1.44	0.75		
			Lung SqCC		24/25				0.41	0.82		
Peng [16]	2015	China	NSCLC	HC	36/36	Serum	qRT-PCR	GAPDH	1.096	0.71	0.89/0.53	6
				HC	120/71				2.0845	0.67	0.99/0.35	
Guo [11]	2015	China	Lung cancer	HC	105/65	Blood	qRT-PCR	GAPDH	10.3444	0.72	0.70/0.60	5
Duan [14]	2016	China	Bladder cancer	HC	120/52	Serum	qRT-PCR	GAPDH	Unclear	0.64	0.57/0.68	6
He [20]	2017	China	Nasopharyngeal carcinoma	HC	101/101	Serum	qRT-PCR	GAPDH	Unclear	0.65	0.66/0.89	6
				Chronic nasopharyngitis	101/20					0.66	0.61/0.85	
				EBV carriers	101/20					0.61	0.53/0.89	

AdCa: adenocarcinoma; AUC: area under curve; GAPDH: Glyceraldehyde-3-phosphate dehydrogenase; HC: Healthy control; SqCC: squamous carcinoma; SCLC: small cell lung carcinoma; NSCLC: non-small cell lung carcinoma; QUADAS: quality assessment for studies of diagnostic accuracy.

**Figure 2:**
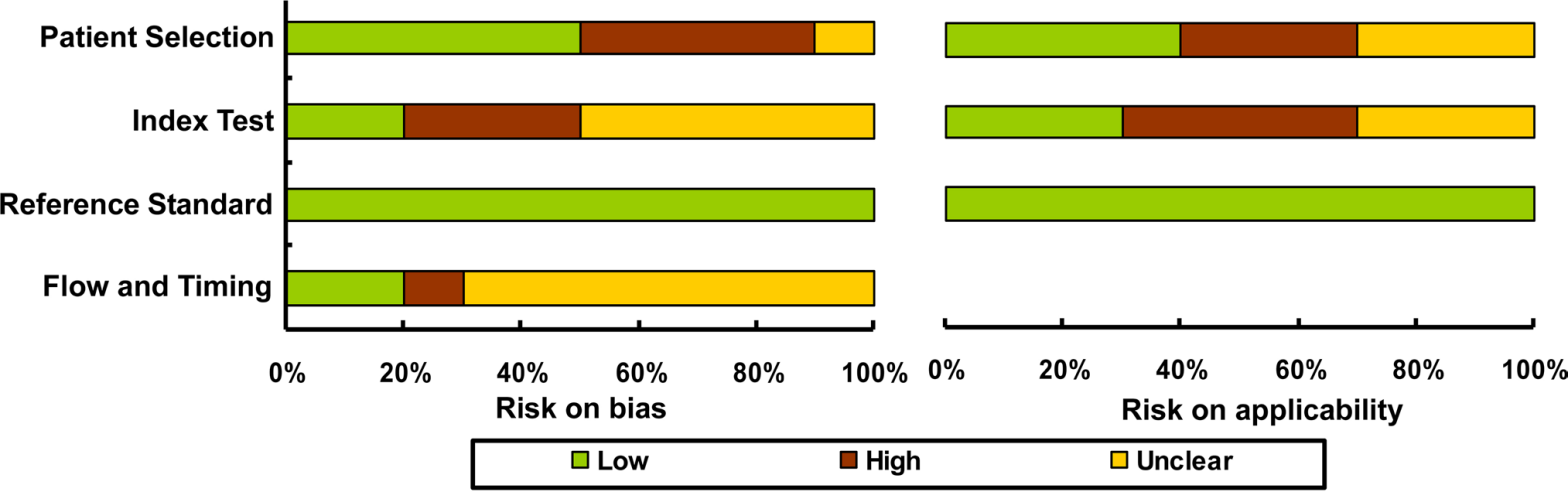
Study quality assessed by the QUADAS-2 checklist

Proportions of risks on bias and applicability by the QUADAS-2 checklist are shown in Figure 2, where all the eligible records showed low risks of bias. Correspondingly, the cumulative scores for the included studies are listed in Table 1, and each study revealed an evaluation score equal or larger than 4, revealing a relatively high quality of all included studies.

### Diagnostic performance

**Figure 3:**
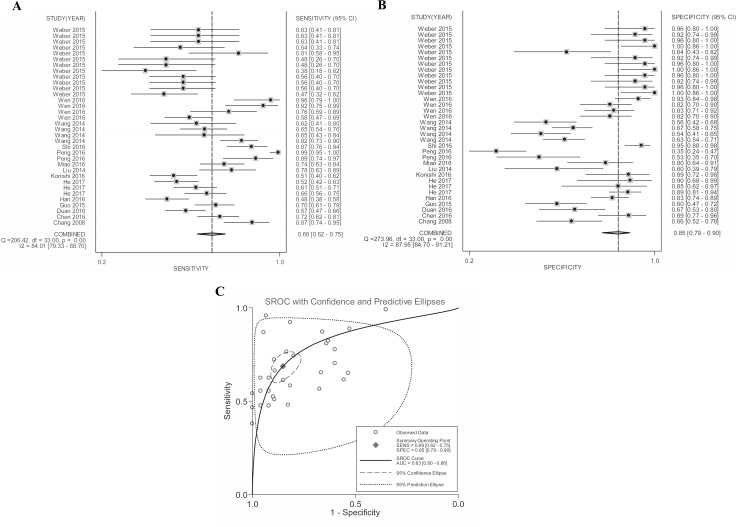
Forest plots of (**A**) pooled sensitivity, (**B**) specificity and (**C**) AUC for the included studies.

Quantitative analysis of the diagnostic performance manifested that elevated *MALAT-1* expression harvested a combined sensitivity of 0.69 (95%CI: 0.62–0.75) and specificity of 0.85 (95%CI: 0.79–0.90) in discriminating cancers from noncancerous controls, corresponding to an AUC value of 0.83 (Figure 3). Moreover, the pooled DOR (diagnostic odds ratio), PLR (positive likelihood ratio) and NLR (negative likelihood ratio) were calculated as 12.56 (95% CI: 8.51–18.55), 4.62 (95% CI: 3.30–6.46) and 0.37 (95% CI: 0.31–0.44), respectively.

### Study heterogeneity

Heterogeneity analysis by *Chi*^2^ and *I*^2^ tests showed obvious heterogeneity across the overall studies (*Q* = 264.95, *P* < 0.001, and *I*^2^ = 99.25%). Additionally, in the subgroup studies by type of carcinoma, test matrix and ethnicity, heterogeneity also appeared in the analyses of pulmonary tumor (*I*^2^ = 99.11%, *P* < 0.001), adenocarcinoma (AdCa) (*I*^2^ = 88.59%, *P* < 0.001), squamous carcinoma (SqCC) (*I*^2^ = 78.66%, *P* = 0.005), gynecologic cancer (*I*^2^ = 74.40%, *P* = 0.020) as well as in serum- (*I*^2^ = 80.20%, *P* < 0.001) and Asian-based (*I*^2^ = 75.90%, *P* < 0.001) analyses (Table 2). Correspondingly, the L’Abbe and Galbraith plots also showed obvious heterogeneity among studies (Supplementary Figure 1).

**Table 2:** Stratified analyses of the diagnostic efficacy of MALAT-1 in cancers

	AUC	Sensitivity (95%CI)	Specificity (95%CI)	DOR (95%CI)	PLR (95%CI)	NLR (95%CI)	Heterogeneity (*I*^2^; *P* value)	Publication bias (*P* value)
Cancer type								
Lung cancer (overall)	0.90	0.71 (0.60–0.81)	0.92 (0.85–0.96)	27.69 (16.08–47.67)	8.67 (4.98–15.07)	0.31 (0.22–0.44)	99.11%; 0.000	0.434
NSCLC	0.88	0.74 (0.68–0.78)	0.68 (0.61–0.74)	18.99 (9.19–39.23)	3.98 (1.78–8.88)	0.41 (0.28–0.59)	0.00%; 0.559	0.126
Lung adenocarcinoma	0.83	0.60 (0.42–0.76)	0.91 (0.76–0.97)	2.77 (1.87–3.67)	7.02 (2.71–18.13)	0.44 (0.30–0.64)	88.59%; 0.000	0.880
Lung squamous carcinoma	0.91	0.68 (0.53–0.80)	0.94 (0.85–0.98)	35.61 (13.95–90.93)	11.99 (4.80–29.97)	0.34 (0.22–0.51)	78.66%; 0.005	0.415
Prostate cancer	0.64	0.72 (0.63–0.79)	0.61 (0.56–0.66)	4.08 (2.53–6.58)	1.86 (1.55–2.23)	0.46 (0.33–0.63)	0.00%; 0.260	0.277
Nasopharyngeal carcinoma	0.84	0.60 (0.54–0.66)	0.88 (0.77–0.95)	11.33 (4.97–25.83)	5.02 (2.49–10.12)	0.46 (0.37–0.56)	0.00%; 0.784	/
Digestive system tumor	0.84	0.67 (0.60–0.74)	0.71 (0.61–0.79)	8.55 (4.51–16.19)	2.46 (1.73–3.52)	0.36 (0.17–0.75)	0.00%; 0.468	0.087
Gynecologic cancer	0.84	0.62 (0.56–0.67)	0.83 (0.77–0.88)	7.97 (3.08–20.66)	3.35 (2.02–5.58)	0.44 (0.26–0.73)	74.40%; 0.020	0.528
Test matrix								
Plasma	0.88	0.62 (0.58–0.66)	0.89 (0.86–0.92)	17.83 (11.54–27.53)	5.93 (4.20–8.37)	0.44 (0.38–0.52)	22.80%; 0.189	0.202
Serum	0.85	0.71 (0.67–0.74)	0.71 (0.66–76)	13.28 (5.22–33.79)	3.21 (1.87–5.51)	0.35 (0.23–0.51)	80.20%; 0.000	0.334
Tissue	0.77	0.63 (0.56–0.70)	0.77 (0.70–0.84)	5.43 (2.78–10.61)	2.35 (1.71–3.23)	0.39 (0.18–0.81)	29.40%; 0.242	0.296
Urine	0.65	0.72 (0.65–0.78)	0.61 (0.56–0.66)	3.64 (1.95–6.77)	1.78 (1.41–2.25)	0.50 (0.34–0.73)	62.2%; 0.047	0.193
Ethnicity								
Asian	0.82	0.69 (0.67–0.71)	0.72 (0.69–0.74)	9.02 (5.85–13.89)	2.84 (2.24–3.61)	0.40 (0.33–0.48)	75.90%; 0.000	0.525
Caucasian	0.82	0.55 (0.50–0.60)	0.93 (0.90–0.96)	19.23 (10.92–33.88)	9.00 (4.45–18.19)	0.49 (0.44–0.55)	0.00%; 0.936	0.371

NSCLC: non-small cell lung carcinoma; CI: confidence interval; PLR: positive likelihood ratio; NLR: negative likelihood ratio; DOR: diagnostic odds ratio.

### Stratified analysis

Due to the existence of significant heterogeneity across the whole analyses, subgroups were analyzed depending on cancer type, test matrix and ethnicity. As exemplified in Table 2, *MALAT-1* testing achieved a high AUC value of 0.90 in the diagnosis of pulmonary tumor (overall), especially in confirming the subtypes of SqCC (AUC = 0.91) and non-small-cell lung cancer (NSCLC) (AUC = 0.88). Of note, the pooled specificities in pulmonary tumor, AdCa and SqCC were shown to be 0.92 (95% CI: 0.85–0.96), 0.91 (95% CI: 0.76–0.97) and 0.94 (95% CI: 0.85–0.98), respectively. Moreover, the diagnostic efficacy of *MALAT-1* in other cancers, including digestive system tumor, gynecologic cancer and nasopharyngeal carcinoma also revealed robust results (Table 2). On the other hand, stratified analyses in terms of test matrix evidenced that plasma-based *MALAT-1* testing presented an AUC of 0.88 better than that of serum- (AUC = 0.85), tissue- (AUC = 0.77) and urine-based (AUC = 0.65) analyses, indicating that plasma might be a suitable test matrix for the analysis of *MALAT-1*. Additionally, testing depending on ethnicity displayed that Asian- and Caucasian-based *MALAT-1* analysis retained equal AUC values (0.82), whereas Caucasian-based analysis harbored a superior specificity of 0.93 (95% CI: 0.90–0.96) and DOR of 19.23 (95% CI: 10.92–33.88) (Table 2).

### Sensitivity analysis and meta-regression

**Figure 4:**
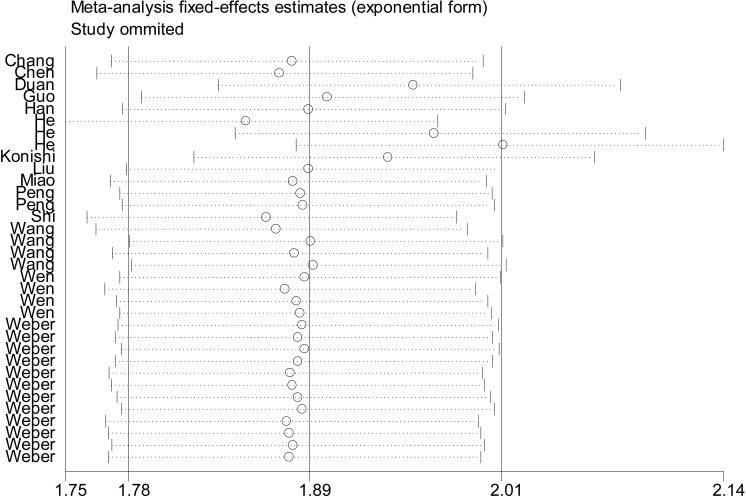
Outlier detection analysis of the overall pooled studies by the fixed-effects estimates

Sensitivity analyses of the overall pooled effect size showed that 1 individual study by He et al. [20] was estimated to be out of the effective line (Figure 4), and its removal resulted in a decrease of heterogeneity in sensitivity (*I*^2^ from 84.01% to 83.85%), but an increase of heterogeneity in specificity (*I*^2^ from 87.95% to 88.22%). Besides that, the pooled NLR dropped from 0.37 to 0.35, and DOR elevated form 12.56 to 12.71. Further univariate meta-regression test was undertaken depending on the covariates of cancer type, specimen source, sample size, reference gene and study quality [23]. As summarized in Table 3, the results showed that different specimen type (RDOR = 0.67, *P* = 0.0012) is more like to be a cause of study heterogeneity.

**Table 3:** Analysis of potential sources of heterogeneity among studies by meta-regression test

Study characteristic	*P*-value	RDOR	95% CI
Cancer type	0.3952	0.95	(0.83–1.08)
Specimen type (plasma vs. serum vs. urine vs. tissue)	0.0012	0.67	(0.54–0.84)
Patient size (< 100 vs. ≥ 100)	0.8093	1.15	(0.34–3.89)
Control size (< 100 vs. ≥ 100)	0.8780	0.91	(0.27–3.09)
Ethnicity ( Asian vs. Caucasian)	0.2137	3.03	(0.51–18.16)
Reference gene ( GAPDH vs. β-actin vs. others)	0.4755	0.92	(0.71–1.18)
Study quality (QUADAS score)	0.5175	0.93	(0.74–1.17)

RDOR: relative diagnostic odds ratio; QUADAS: quality assessment for studies of diagnostic accuracy; GAPDH: glyceraldehyde-phosphate dehydrogenase; CI: confidence interval; vs.:versus.

### Publication bias

**Figure 5:**
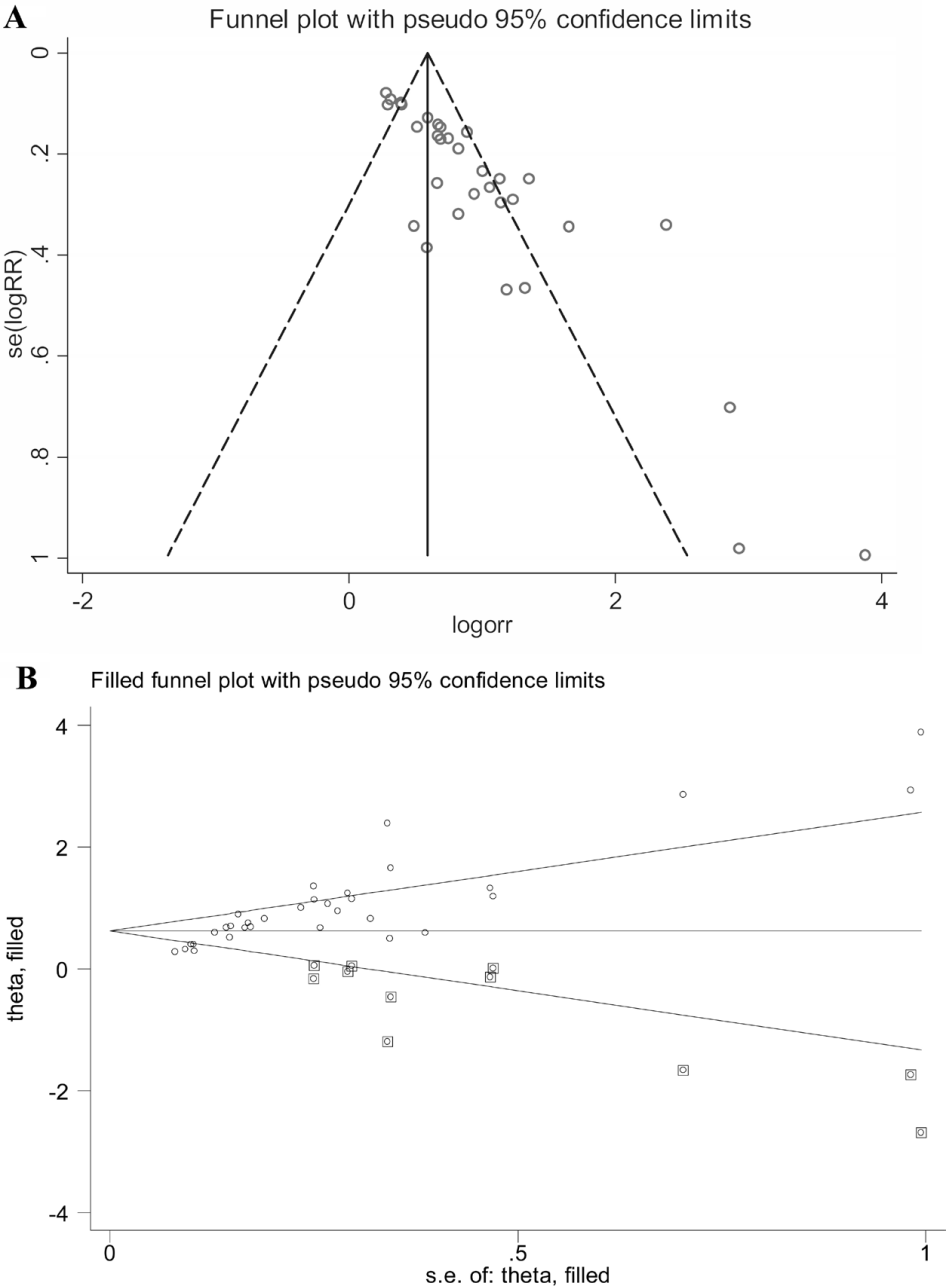
Publication bias assessed by Funnel plot (**A**) and “Duval and Tweedie nonparametric trim and fill method” (**B**). Hollow cycle in box represents the estimated missing study.

Publication bias was tested with Funnel plot and Deeks’ funnel plot asymmetry test. For the overall pooled effect size, both visual Funnel plot (Figure 5A) and quantitative Deeks’ funnel plot asymmetry test (Supplementary Figure 2, *P* < 0.05) displayed obvious publication bias among studies. In consequence, the “Duval and Tweedie nonparametric trim and fill method” was employed to elucidate the possible effects of bias on the pooled analysis [24]. As shown in Figure 5B, the imputed analyses generated a symmetrical funnel plot after filling the hypothetical 12 missing studies. Moreover, the linear trimming and filling estimator showed an estimate variance of 0.111 (*P* < 0.001) before adjustment versus that of 0.166 (*P* < 0.001) after adjustment, suggesting that the pooled effect was slightly altered before and after adjustments. Evaluation of the publication bias in the subgroup studies was performed as well and no clear bias was detected among stratified analyses (Table 2).

## DISCUSSION

*MALAT-1* is a kind of non-protein-coding RNA transcripts, and its elevated expression status has been demonstrated to be implicated in the occurrence and development of various carcinomas [5, 6]. The diagnostic feature of *MALAT-1* in cancers has been documented by many single studies [7–20]. However, accuracies from single studies are often compromised due to limited sample size and single-center design. In the current study, we seek to conduct a systematic meta-analysis to elucidate the global diagnostic efficacy of *MALAT-1* in human cancers.

Our data showed that *MALAT-1* expression profiling sustained a pooled sensitivity of 0.69, specificity of 0.85 and AUC of 0.83 in discriminating cancer patients from noncancerous controls, revealing an overall high efficacy for the overall diagnostic test. Moreover, the pooled DOR of 12.56 also showed a powerful capability of *MALAT-1* testing in discriminating cancers from cancer-free individuals [25]. The likelihood ratios involved PLR (positive likelihood ratio) and NLR (negative likelihood ratio) are often utilized for assessing the value of performing a diagnostic test [26]. In our study, the estimated PLR value of 4.62 means that the probability of cancer cases yield *MALAT-1* testing positive is nearly 5-fold higher towards the cases do not have *MALAT-1* testing positive; likewise, the NLR of 0.37 means that the probability of cancer cases retain *MALAT-1* testing negative is only 0.37-fold towards the cases do not have *MALAT-1* testing negative. All these data indicated that elevated *MALAT-1* achieves a powerful efficacy to aid in the diagnosis of cancers.

In the present study, heterogeneity seems to be existed among the overall pooled analyses mainly due to the included different types of cancers. In consequence, we further conducted subgroup studies depending on the type of carcinoma, test matrix and ethnicity. Our results revealed that elevated *MALAT-1* expression achieved a high AUC value of 0.90 in the diagnosis of pulmonary tumor as well as the subtypes of SqCC (AUC of 0.91) and NSCLC (AUC of 0.88). Importantly, *MALAT-1* testing showed promising specificities higher than 0.90 in confirming lung cancer (overall), as well as the AdCa and SqCC subtype. Analysis of the efficacy in other types manifested that *MALAT-1* testing harvested an AUC of 0.84, specificity of 0.88 and DOR of 11.33 in identifying nasopharyngeal carcinoma, suggesting that *MALAT-1* might be developed as a promising biomarker for nasopharyngeal carcinoma as well. Besides that, *MALAT-1* showed equal AUC values of 0.84 in both of the digestive system and gynecologic tumors, but a higher combined specificity of 0.83 was observed in the latter.

The matrix differences of lncRNA signature in gastric cancer have been confirmed by our previous study [23]. In supporting with the previous findings, the current analysis found that plasma-based *MALAT-1* testing presented a better AUC value than the serum-, tissue- and urine-based analyses, indicating that plasma might be a suitable matrix for the analysis of *MALAT-1* expression in cancers. On the other hand, our subgroup analysis by region showed that Asian- and Caucasian-based *MALAT-1* testing sustained equal AUC values (0.82), whereas the latter harbored a superior specificity (0.93) and DOR (19.23). Notwithstanding, the Caucasian-based analysis were only from 1 study (by Weber et al. [8]), thus, more evidences are warranted to confirm this point.

On the other hand, due to the existence of significant heterogeneity across the whole effect size, we further conducted sensitivity analysis and meta-regression test. The influence analysis identified 1 outlier study [20], and its removal resulted in a decrease of heterogeneity in sensitivity but an elevation of heterogeneity in specificity, hinting that included outlier study is a factor that contributing to the generation of heterogeneities. Moreover, univariate meta-regression test showed that different specimen type is more like to be another cause of heterogeneity among studies. We also observed significant publication bias in the overall pooled analysis. To deeply assess the possible impacts of publication bias on our pooled effects, the Duval and Tweedie nonparametric trim and fill procedure was undertaken [24]. The imputed analyses identified 12 missing studies, and after filling with the 12 missing hypothetical studies, the adjusted effect was slightly altered as compared with the unadjusted one, indicating that the overall pooled accuracy does not yield to the impacts from publication bias.

In summary, our findings provide evidence that elevated *MALAT-1* appeared to be a potential diagnostic marker for patients with cancer and could be rated as an auxiliary marker to aid in cancer diagnosis. Nevertheless, our study still reveals several limitations: Firstly, the analysis may have bias in some cancer types that analyzed based on small sample sizes. Secondly, the sample type, control sources as well as the reference gene for testing are complicated. Lastly, most of the included studies are conducted in Asian, and there might be ethnicity bias in the overall combined effects. Further comprehensive and large-scale studies are still warranted to confirm our evidence.

## MATERIALS AND METHODS

### Search strategy

The international databases included PubMed/Medline, Evidence-Based Medicine Reviews, EBSCO, and BioMed Central were searched for the retrieval of eligible articles in English, and that CNKI, Wanfang and Weipu databases were retrieved for obtainment of studies published in Chinese. Date of publication was set up to May 1st, 2017. The search approaches with Medical Subject Heading terms or free-text words were utilized as: *(“long non-coding RNA” or “lncRNA” or “MALAT-1” or “Metastasis-Associated-in-Lung-Adenocarcinoma-Transcript-1”) and (“cancer” or “carcinoma” or “tumor” or “neoplasm” or “malignancy”) and/or (“diagnosis” or “area under the curve” or “AUC” or “sensitivity” or “specificity” or “ROC” or “Receiver operation characteristic curve”)*. We also manually searched the attached references in articles to increase search sensitivity.

### Study selection

Studies were firstly included if they in accordance with the following criteria: (1) studies evaluated the diagnostic performance(s) of *MALAT-1* in cancer(s); (2) expression of *MALAT-1* was assessed by quantitative real-time polymerase chain reaction (qRT-PCR) or microarray analysis or other approaches; (3) studies had at least a disease group and a control group, with a sample size larger than 20; (4) the estimated sensitivity, specificity or AUC were available; and (5) the full-text was published in English or Chinese. Studies did not match the following criteria were excluded: (1) the control types were undefined or the sample sizes were smaller than 20; and (2) studies identified as review articles, basic research, animal studies, comments, letters or conference abstracts.

### Study bias assessment

The bias among eligible studies was evaluated in duplicates by two group authors, using the evidence-based Quality Assessment for Studies of Diagnostic Accuracy II (QUADAS-2) checklist ( www.quadas.org) [27]. This evaluation tool comprises four phases: review question, review-specific tailoring, flow diagram, and judgments on bias and applicability. Study quality was mainly based on the judgments of risk on bias and applicability, in which the following sections were included: patient selection, index test, reference standard, flow and timing. Risk of bias can be rated as “low”, “high”, or “unclear”, corresponding to a score of “1”, “0” and “0”. A judgment answer of “high” indicates potential bias existing among studies. Study awarded a cumulative score higher or equal to 4 was considered as eligible and that lower than 4 will be eliminated for the meta-analysis.

### Data extraction

The basic information of articles were extracted and collected in twice by two trained person, including author's name, article publication date, study population, cancer type, sample size and type, control size and type, test method, reference gene, sensitivity, specificity, AUC (area under curve), cut-off value, etc. Any disagreements during data extraction will be solved by group consensus (all group members discussed and solved the disagreements together).

### Statistical analysis

We conducted and reported this systematic meta-analysis in terms of the guidelines of the Preferred Reporting Items for Systematic reviews and Meta-Analyses (PRASMA) statement [28]. All statistics were conducted based on STATA 12.0 program (Stata Corporation, College Station, TX, USA). Heterogeneity from eligible studies was estimated by *Chi*^2^ (Chi-squared), and *I*^2^ (I-squared) tests as well as the L’Abbe and Galbraith plot analysis. Either *P* < 0.05 for the *Chi*^2^ test or *I*^2^ > 50% for the *I*^2^ test were both considered as pooled effects with significant inconsistency. The pooled effect sizes included sensitivity, specificity, PLR, NLR, and DOR were generated using either a random-effect model or a fixed-effect model depending on study heterogeneity. The underlying sources of heterogeneity were further traced by sensitivity analysis and univariate meta-regression test [23]. Study bias due to publication was estimated by visual Funnel plot and quantifiable Deeks’ funnel plot asymmetry test, with a statistical level of *P* < 0.05. The possible effects of publication bias on the overall pooled accuracy were assessed using “Duval and Tweedie nonparametric trim and fill method” [24].

## SUPPLEMENTARY MATERIALS FIGURES


