# Supplementary material for: Diagnostic efficacy of long non-coding RNA MALAT-1 in human cancers: a meta-analysis study
Source: Oncotarget. 2017 Sep 18;8(60):102291–300. doi: 10.18632/oncotarget.21013 (PMC5731954; doi:10.18632/oncotarget.21013)
Supplement: Supplementary file 1 [file oncotarget-08-102291-s001.pdf]

Diagnostic efficacy of long non-coding RNA *MALAT-1* in human cancers: a meta-analysis study

SUPPLEMENTARY MATERIALS

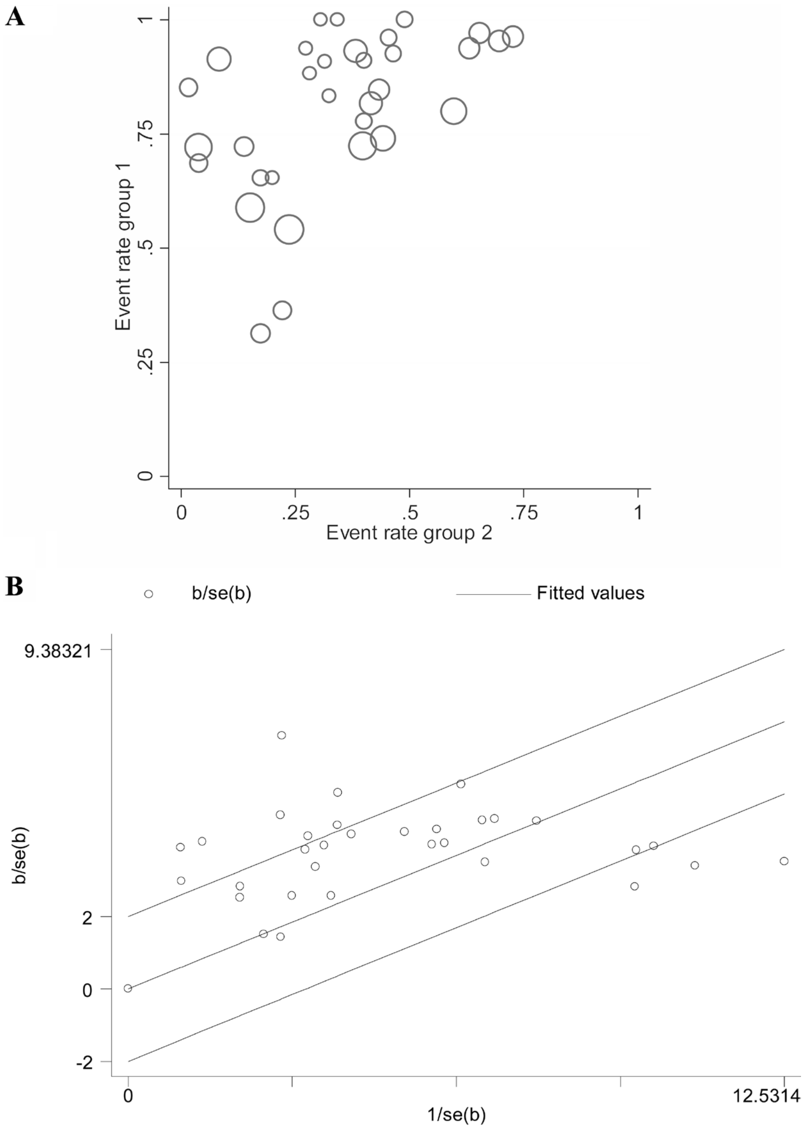

Supplementary Figure 1: Heterogeneity from eligible studies was judged by the L'Abbe plot (**A**) and Galbraith plot analysis (**B**). The L'Abbe plot will present a linear distribution if studies are homogeneous. In the Galbraith plot, if there are studies out of the 95%CI line, the analyses will be regarded as heterogeneous.

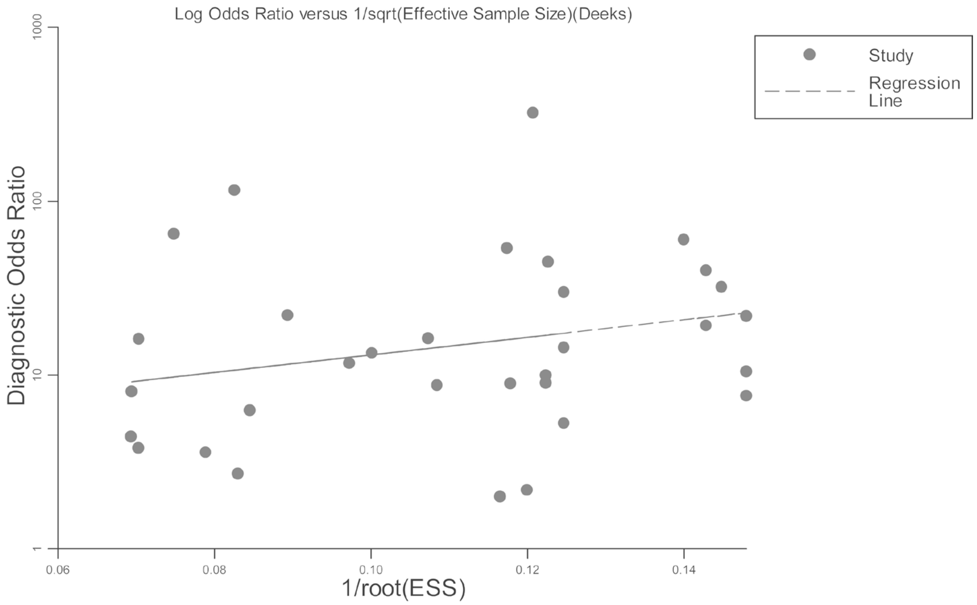

Supplementary Figure 2: Analysis of publication bias of the overall pooled effects by quantified Deeks' funnel plot asymmetry test.
